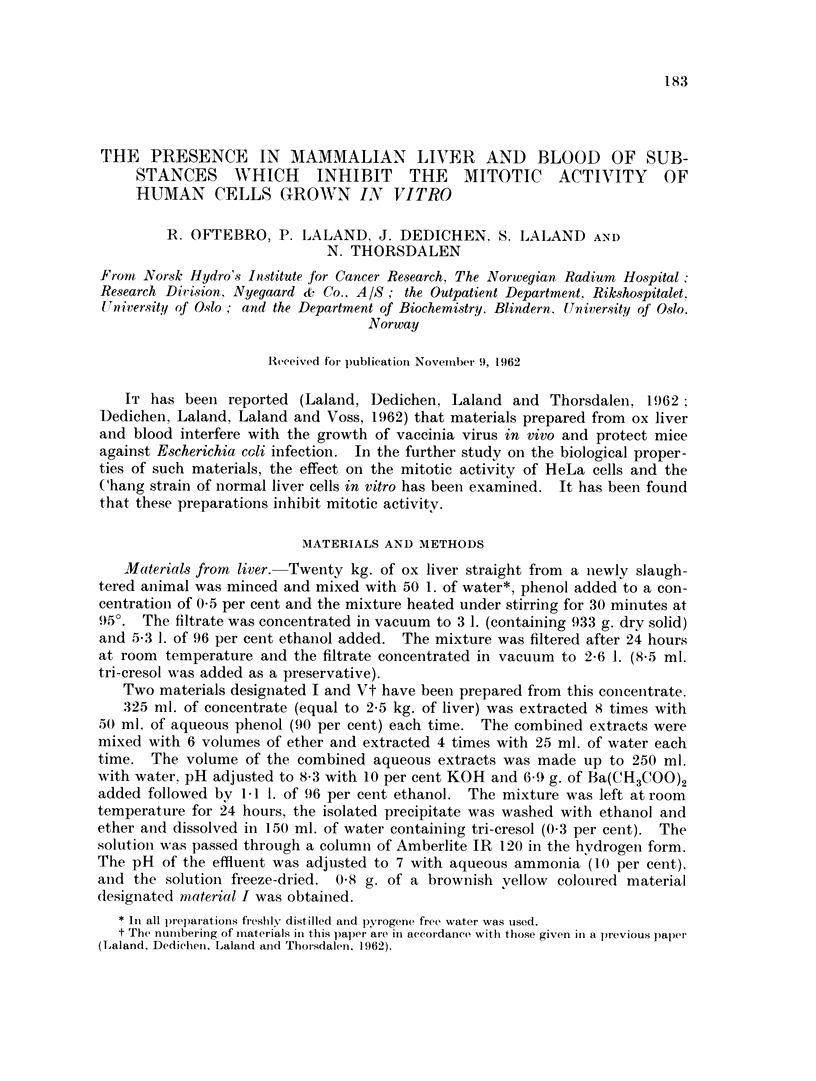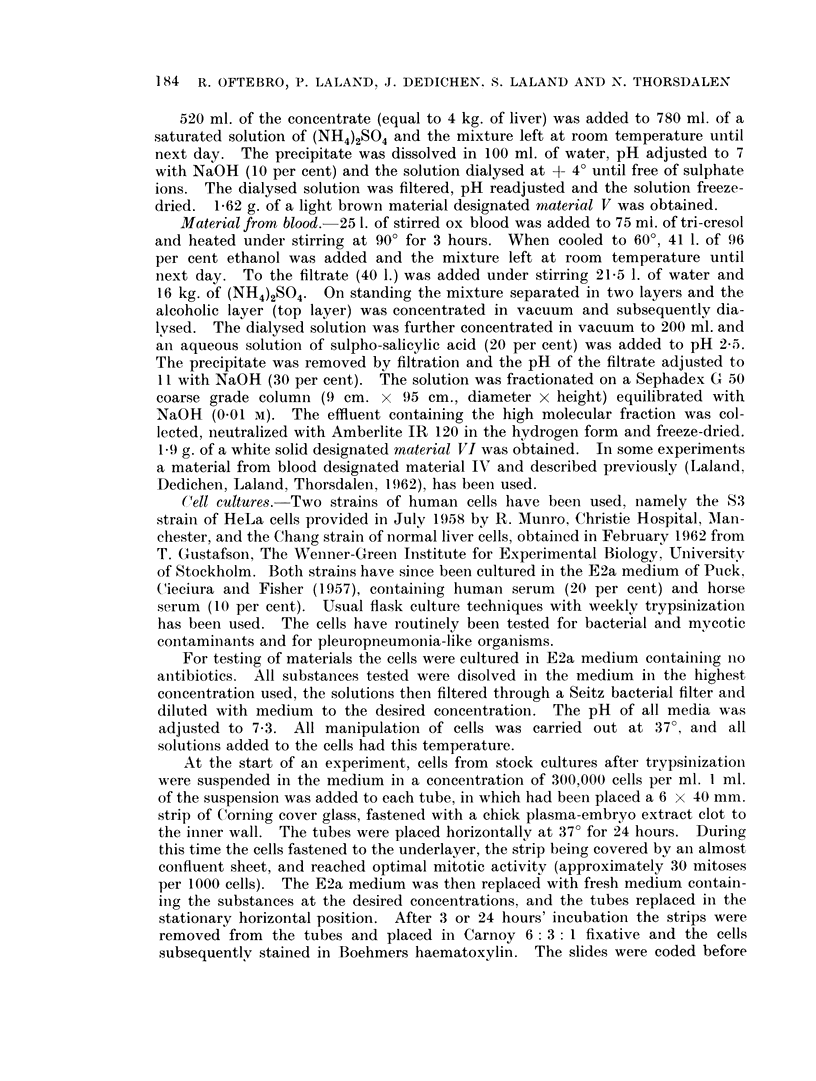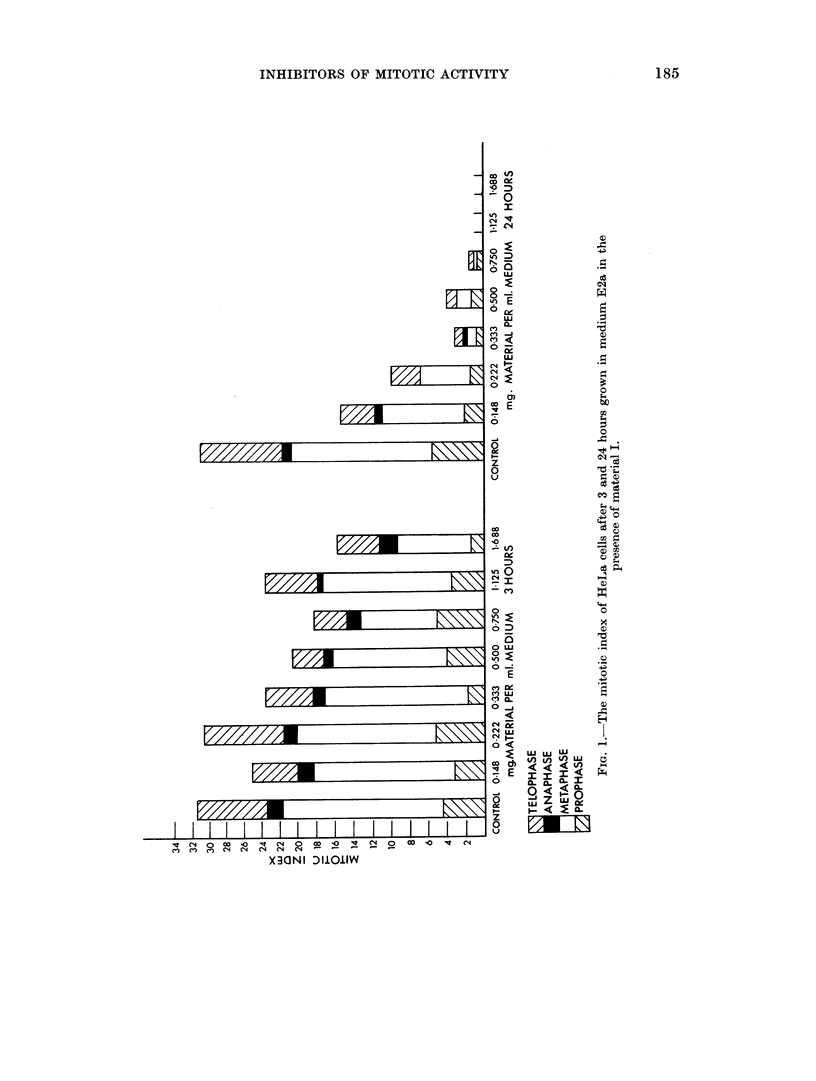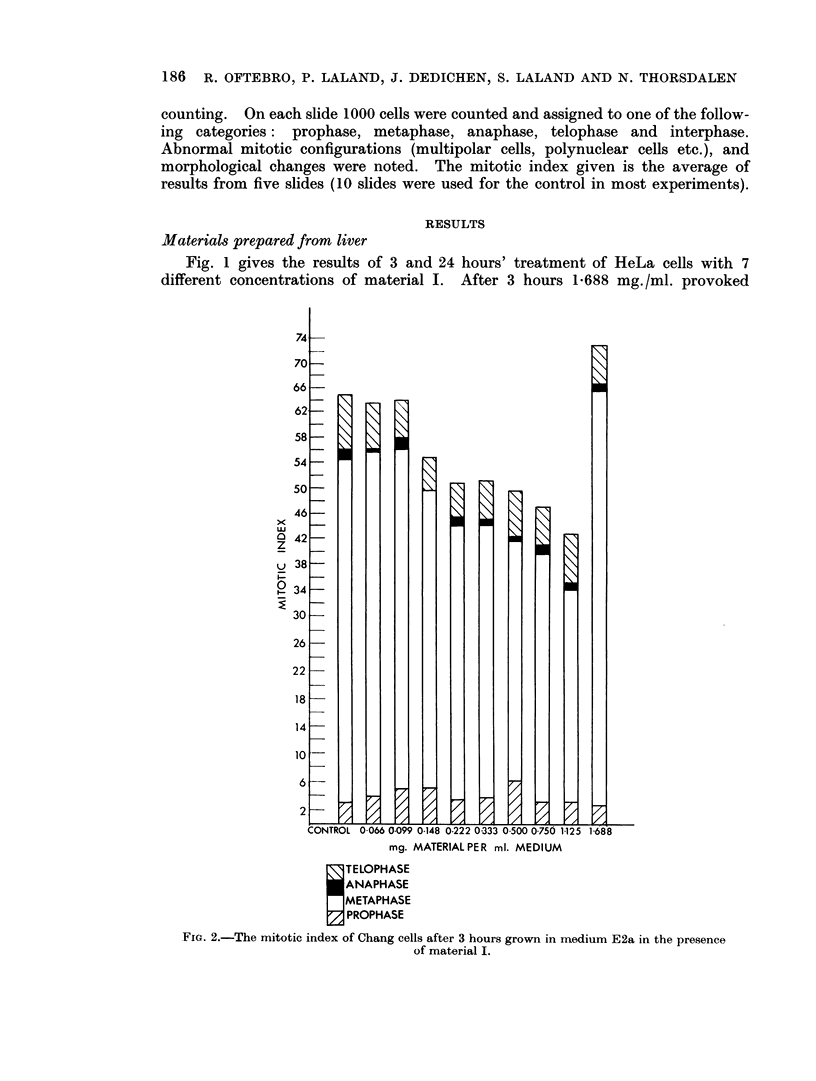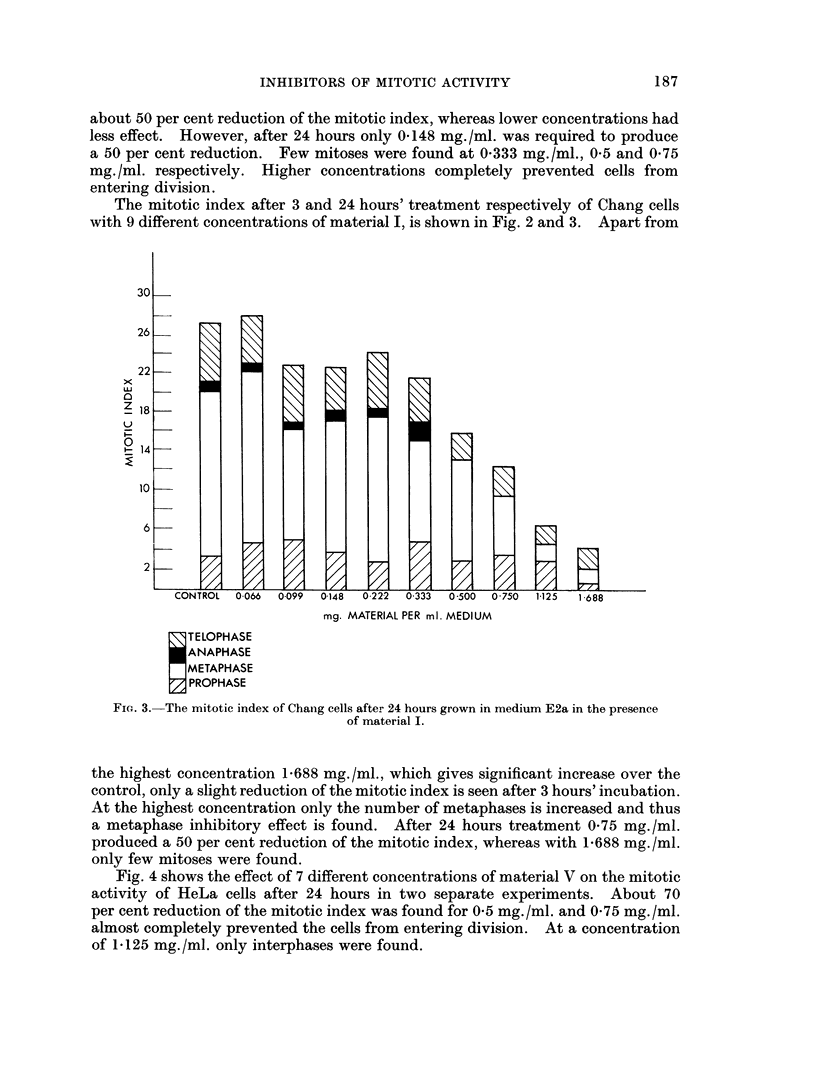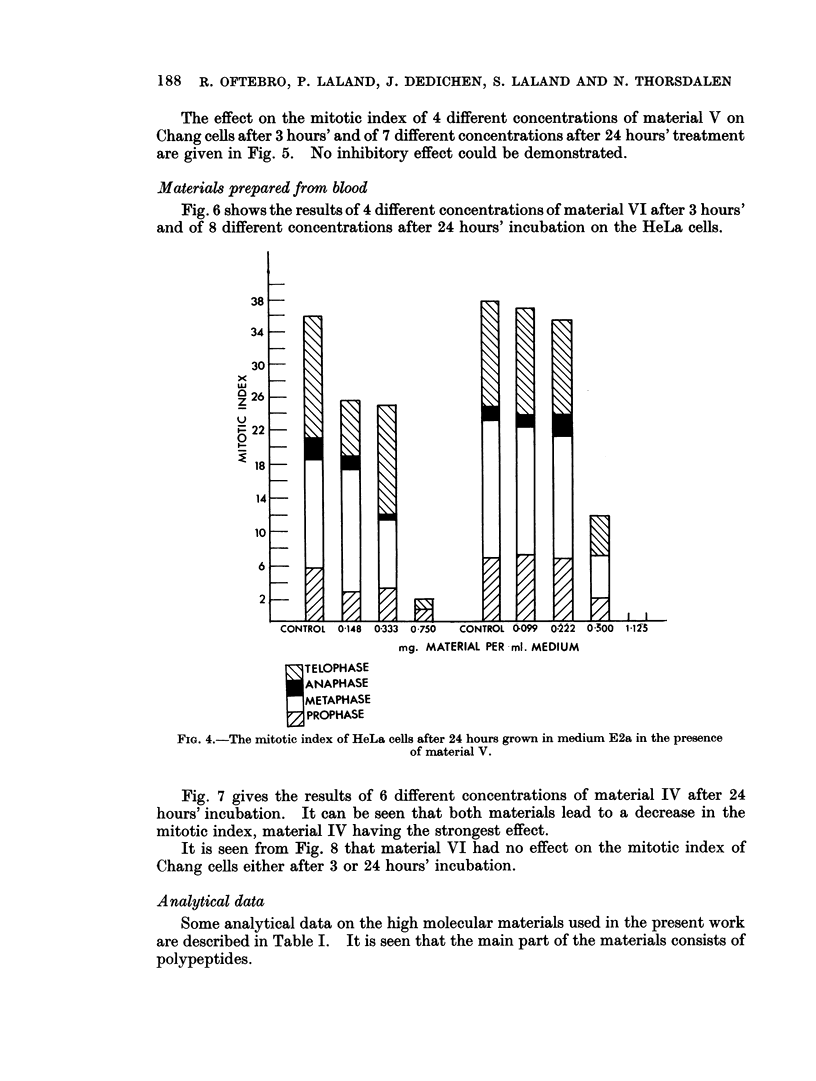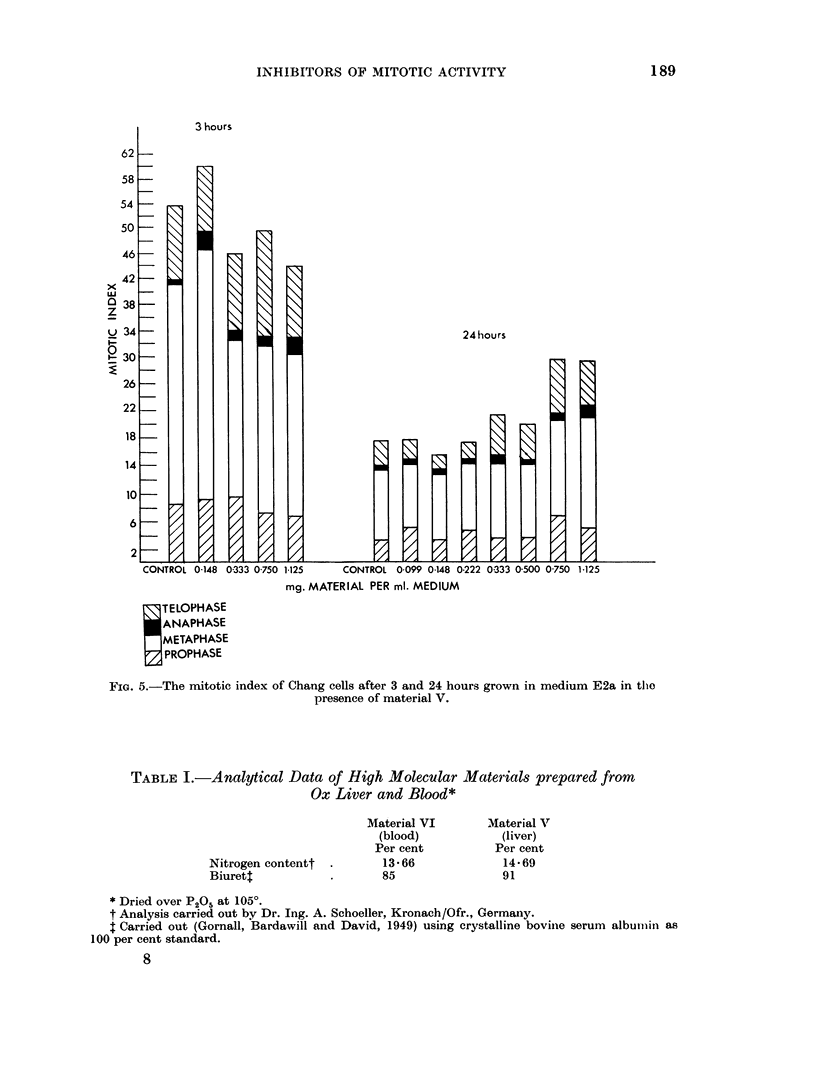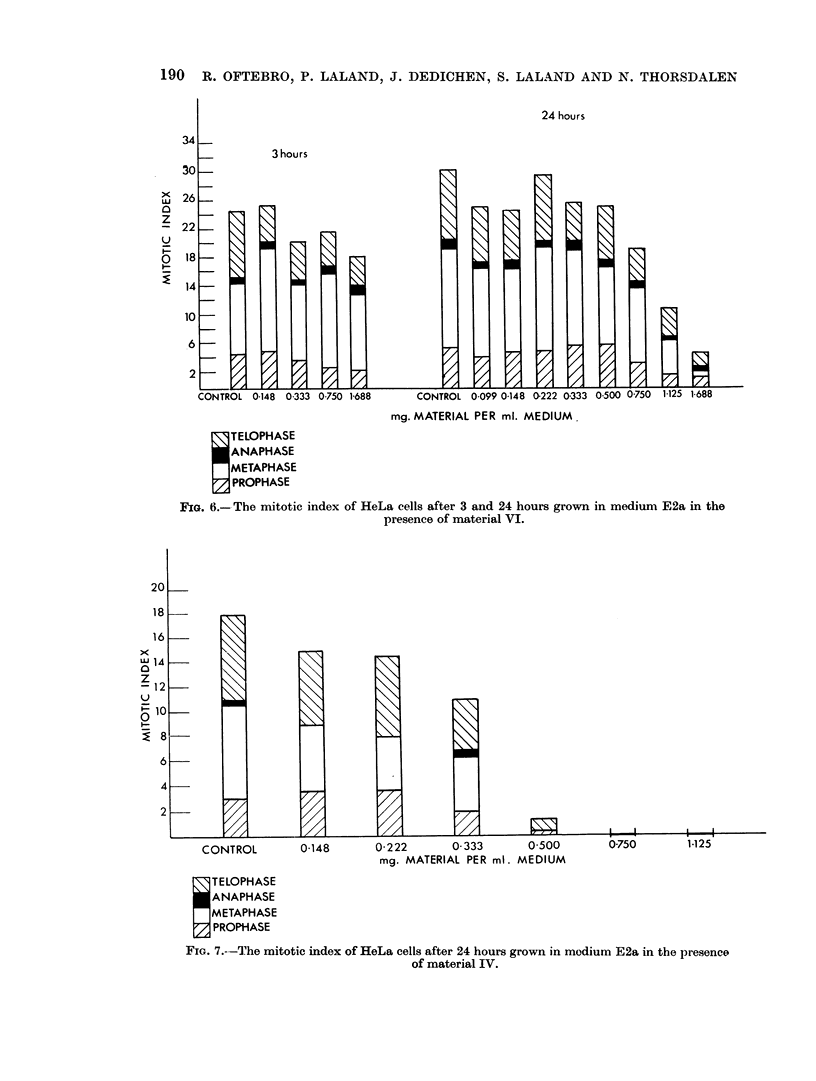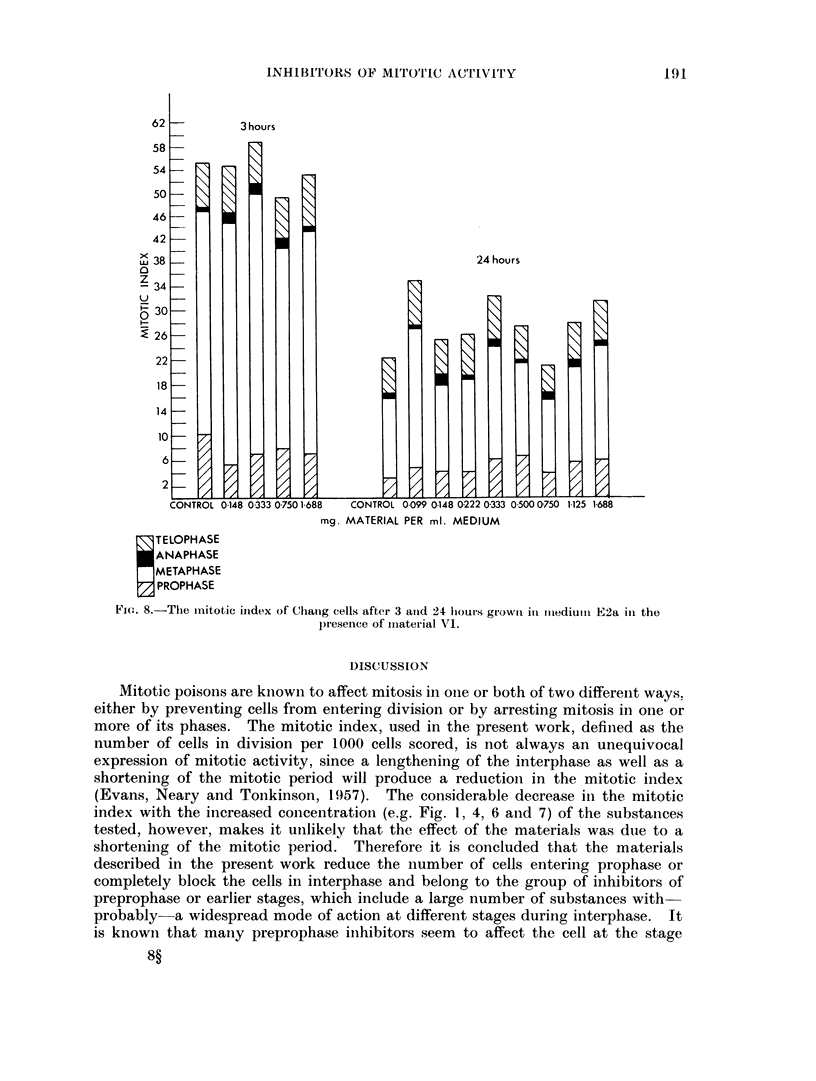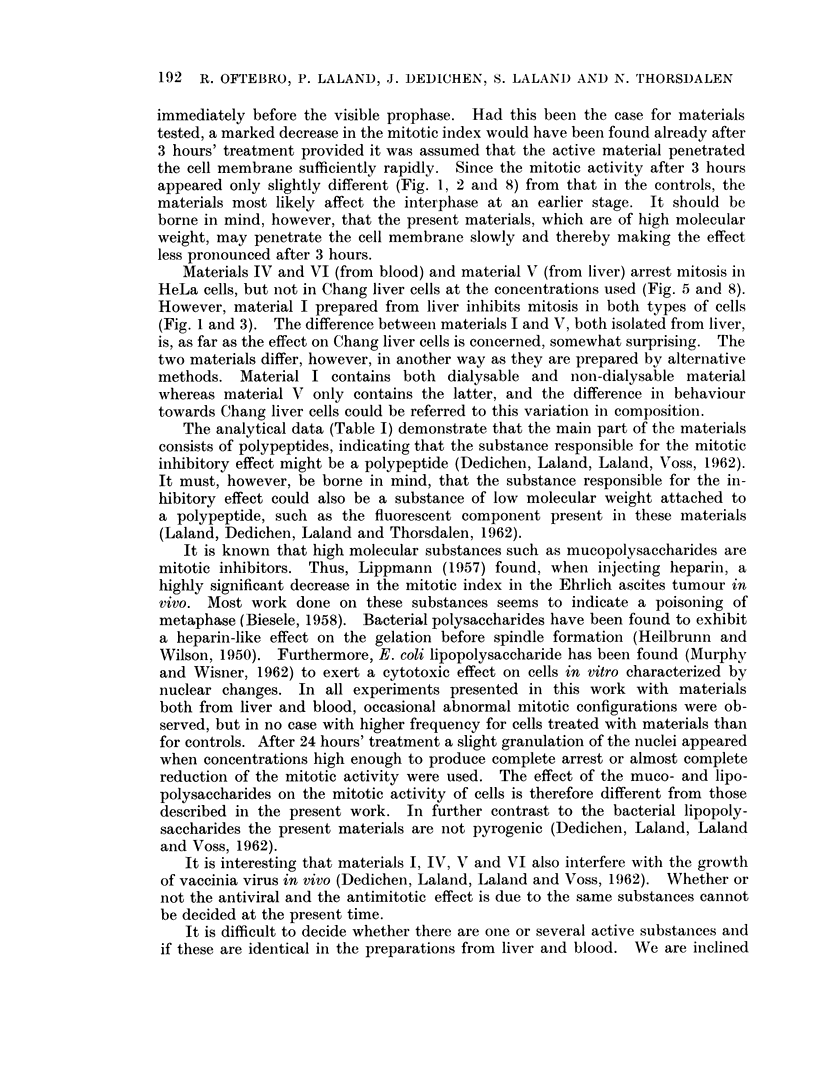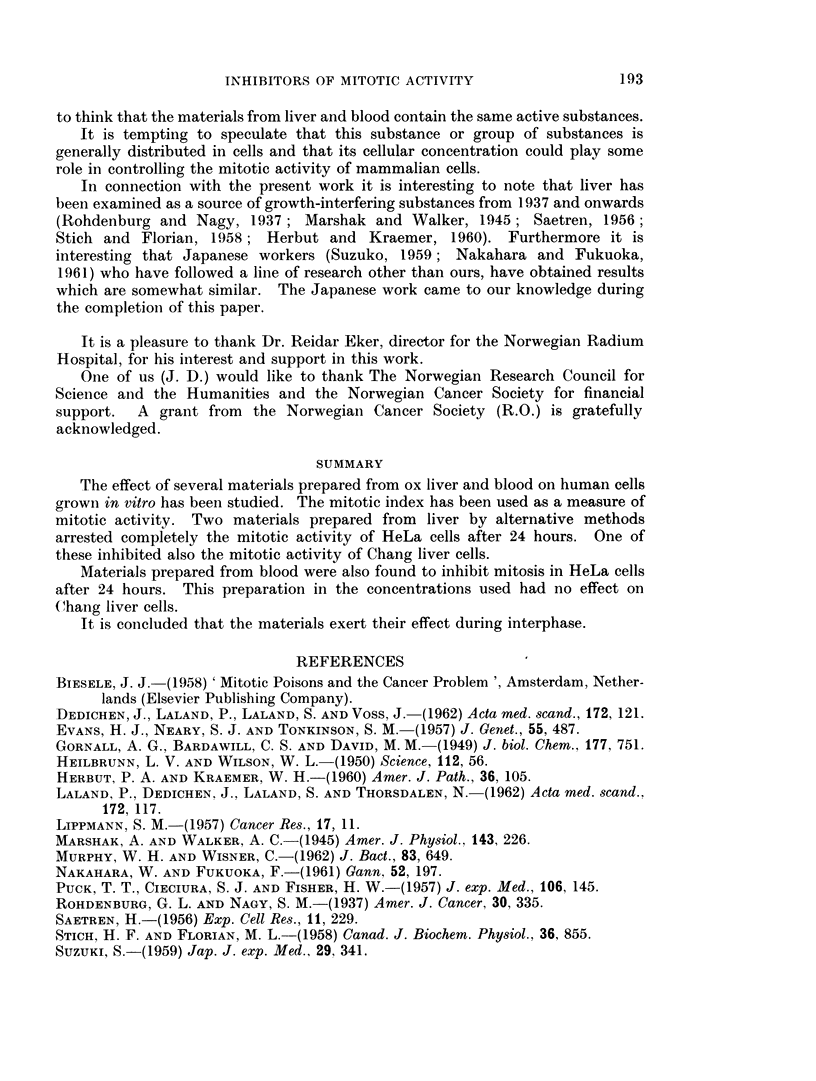# The Presence in Mammalian Liver and Blood of Substances which Inhibit the Mitotic Activity of Human Cells Grown in Vitro

**DOI:** 10.1038/bjc.1963.26

**Published:** 1963-03

**Authors:** R. Oftebro, P. Laland, J. Dedichen, S. Laland, N. Thorsdalen


### THE PRESENCE IN MAMMALIAN LIVER AND BLOOD OF SUBSTANCES WHICH INHIBIT THE MITOTIC ACTIVITY OF HUMAN CELLS GROWN *IN VITRO*

R. OFTEBRO, P. LALAND, J. DEDICHEN, S. LALAND AND N. THORSDALEN

*From Norsk Hydro's Institute for Cancer Research, The Norwegian Radium Hospital; Research Division, Nyegaard & Co., A/S; the Outpatient Department, Rikshospitalet, University of Oslo; and the Department of Biochemistry, Blindern, University of Oslo, Norway*

Received for publication November 9, 1962

It has been reported (Laland, Dedichen, Laland and Thorsdalen, 1962; Dedichen, Laland, Laland and Voss, 1962) that materials prepared from ox liver and blood interfere with the growth of vaccinia virus *in vivo* and protect mice against *Escherichia coli* infection. In the further study on the biological properties of such materials, the effect on the mitotic activity of HeLa cells and the Chang strain of normal liver cells *in vitro* has been examined. It has been found that these preparations inhibit mitotic activity.

### MATERIALS AND METHODS

*Materials from liver.*—Twenty kg. of ox liver straight from a newly slaughtered animal was minced and mixed with 50 l. of water*, phenol added to a concentration of 0·5 per cent and the mixture heated under stirring for 30 minutes at 95°. The filtrate was concentrated in vacuum to 3 l. (containing 933 g. dry solid) and 5·3 l. of 96 per cent ethanol added. The mixture was filtered after 24 hours at room temperature and the filtrate concentrated in vacuum to 2·6 l. (8·5 ml. tri-cresol was added as a preservative).

Two materials designated I and V† have been prepared from this concentrate.

325 ml. of concentrate (equal to 2·5 kg. of liver) was extracted 8 times with 50 ml. of aqueous phenol (90 per cent) each time. The combined extracts were mixed with 6 volumes of ether and extracted 4 times with 25 ml. of water each time. The volume of the combined aqueous extracts was made up to 250 ml. with water, pH adjusted to 8·3 with 10 per cent KOH and 6·9 g. of $Ba(CH_3COO)_2$ added followed by 1·1 l. of 96 per cent ethanol. The mixture was left at room temperature for 24 hours, the isolated precipitate was washed with ethanol and ether and dissolved in 150 ml. of water containing tri-cresol (0·3 per cent). The solution was passed through a column of Amberlite IR 120 in the hydrogen form. The pH of the effluent was adjusted to 7 with aqueous ammonia (10 per cent), and the solution freeze-dried. 0·8 g. of a brownish yellow coloured material designated *material I* was obtained.

* In all preparations freshly distilled and pyrogene free water was used.
† The numbering of materials in this paper are in accordance with those given in a previous paper (Laland, Dedichen, Laland and Thorsdalen, 1962).

520 ml. of the concentrate (equal to 4 kg. of liver) was added to 780 ml. of a saturated solution of $(NH_4)_2SO_4$ and the mixture left at room temperature until next day. The precipitate was dissolved in 100 ml. of water, pH adjusted to 7 with NaOH (10 per cent) and the solution dialysed at + 4° until free of sulphate ions. The dialysed solution was filtered, pH readjusted and the solution freeze-dried. 1·62 g. of a light brown material designated *material V* was obtained.

*Material from blood.*—25 l. of stirred ox blood was added to 75 ml. of tri-cresol and heated under stirring at 90° for 3 hours. When cooled to 60°, 41 l. of 96 per cent ethanol was added and the mixture left at room temperature until next day. To the filtrate (40 l.) was added under stirring 21·5 l. of water and 16 kg. of $(NH_4)_2SO_4$. On standing the mixture separated in two layers and the alcoholic layer (top layer) was concentrated in vacuum and subsequently dialysed. The dialysed solution was further concentrated in vacuum to 200 ml. and an aqueous solution of sulpho-salicylic acid (20 per cent) was added to pH 2·5. The precipitate was removed by filtration and the pH of the filtrate adjusted to 11 with NaOH (30 per cent). The solution was fractionated on a Sephadex G 50 coarse grade column (9 cm. × 95 cm., diameter × height) equilibrated with NaOH (0·01 M). The effluent containing the high molecular fraction was collected, neutralized with Amberlite IR 120 in the hydrogen form and freeze-dried. 1·9 g. of a white solid designated *material VI* was obtained. In some experiments a material from blood designated material IV and described previously (Laland, Dedichen, Laland, Thorsdalen, 1962), has been used.

*Cell cultures.*—Two strains of human cells have been used, namely the S3 strain of HeLa cells provided in July 1958 by R. Munro, Christie Hospital, Manchester, and the Chang strain of normal liver cells, obtained in February 1962 from T. Gustafson, The Wenner-Green Institute for Experimental Biology, University of Stockholm. Both strains have since been cultured in the E2a medium of Puck, Cieciura and Fisher (1957), containing human serum (20 per cent) and horse serum (10 per cent). Usual flask culture techniques with weekly trypsinization has been used. The cells have routinely been tested for bacterial and mycotic contaminants and for pleuropneumonia-like organisms.

For testing of materials the cells were cultured in E2a medium containing no antibiotics. All substances tested were disolved in the medium in the highest concentration used, the solutions then filtered through a Seitz bacterial filter and diluted with medium to the desired concentration. The pH of all media was adjusted to 7·3. All manipulation of cells was carried out at 37°, and all solutions added to the cells had this temperature.

At the start of an experiment, cells from stock cultures after trypsinization were suspended in the medium in a concentration of 300,000 cells per ml. 1 ml. of the suspension was added to each tube, in which had been placed a 6 × 40 mm. strip of Corning cover glass, fastened with a chick plasma-embryo extract clot to the inner wall. The tubes were placed horizontally at 37° for 24 hours. During this time the cells fastened to the underlayer, the strip being covered by an almost confluent sheet, and reached optimal mitotic activity (approximately 30 mitoses per 1000 cells). The E2a medium was then replaced with fresh medium containing the substances at the desired concentrations, and the tubes replaced in the stationary horizontal position. After 3 or 24 hours' incubation the strips were removed from the tubes and placed in Carnoy 6 : 3 : 1 fixative and the cells subsequently stained in Boehmers haematoxylin. The slides were coded before

FIG. 1.—The mitotic index of HeLa cells after 3 and 24 hours grown in medium E2a in the presence of material I.

counting. On each slide 1000 cells were counted and assigned to one of the following categories: prophase, metaphase, anaphase, telophase and interphase. Abnormal mitotic configurations (multipolar cells, polynuclear cells etc.), and morphological changes were noted. The mitotic index given is the average of results from five slides (10 slides were used for the control in most experiments).

### RESULTS

*Materials prepared from liver*

Fig. 1 gives the results of 3 and 24 hours' treatment of HeLa cells with 7 different concentrations of material I. After 3 hours 1·688 mg./ml. provoked

Fig. 2.—The mitotic index of Chang cells after 3 hours grown in medium E2a in the presence of material I.

about 50 per cent reduction of the mitotic index, whereas lower concentrations had less effect. However, after 24 hours only 0·148 mg./ml. was required to produce a 50 per cent reduction. Few mitoses were found at 0·333 mg./ml., 0·5 and 0·75 mg./ml. respectively. Higher concentrations completely prevented cells from entering division.

The mitotic index after 3 and 24 hours' treatment respectively of Chang cells with 9 different concentrations of material I, is shown in Fig. 2 and 3. Apart from

FIG. 3.—The mitotic index of Chang cells after 24 hours grown in medium E2a in the presence of material I.

the highest concentration 1·688 mg./ml., which gives significant increase over the control, only a slight reduction of the mitotic index is seen after 3 hours' incubation. At the highest concentration only the number of metaphases is increased and thus a metaphase inhibitory effect is found. After 24 hours treatment 0·75 mg./ml. produced a 50 per cent reduction of the mitotic index, whereas with 1·688 mg./ml. only few mitoses were found.

Fig. 4 shows the effect of 7 different concentrations of material V on the mitotic activity of HeLa cells after 24 hours in two separate experiments. About 70 per cent reduction of the mitotic index was found for 0·5 mg./ml. and 0·75 mg./ml. almost completely prevented the cells from entering division. At a concentration of 1·125 mg./ml. only interphases were found.

The effect on the mitotic index of 4 different concentrations of material V on Chang cells after 3 hours' and of 7 different concentrations after 24 hours' treatment are given in Fig. 5. No inhibitory effect could be demonstrated.

*Materials prepared from blood*

Fig. 6 shows the results of 4 different concentrations of material VI after 3 hours' and of 8 different concentrations after 24 hours' incubation on the HeLa cells.

FIG. 4.—The mitotic index of HeLa cells after 24 hours grown in medium E2a in the presence of material V.

Fig. 7 gives the results of 6 different concentrations of material IV after 24 hours' incubation. It can be seen that both materials lead to a decrease in the mitotic index, material IV having the strongest effect.

It is seen from Fig. 8 that material VI had no effect on the mitotic index of Chang cells either after 3 or 24 hours' incubation.

*Analytical data*

Some analytical data on the high molecular materials used in the present work are described in Table I. It is seen that the main part of the materials consists of polypeptides.

FIG. 5.—The mitotic index of Chang cells after 3 and 24 hours grown in medium E2a in the presence of material V.

TABLE I.—*Analytical Data of High Molecular Materials prepared from Ox Liver and Blood**

| | Material VI (blood) Per cent | Material V (liver) Per cent |
|---|---|---|
| Nitrogen content† . | 13·66 | 14·69 |
| Biuret‡ . | 85 | 91 |

* Dried over $P_2O_5$ at 105°.

† Analysis carried out by Dr. Ing. A. Schoeller, Kronach/Ofr., Germany.

‡ Carried out (Gornall, Bardawill and David, 1949) using crystalline bovine serum albumin as 100 per cent standard.

Fig. 6.—The mitotic index of HeLa cells after 3 and 24 hours grown in medium E2a in the presence of material VI.

Fig. 7.—The mitotic index of HeLa cells after 24 hours grown in medium E2a in the presence of material IV.

Fig. 8.—The mitotic index of Chang cells after 3 and 24 hours grown in medium E2a in the presence of material VI.

### DISCUSSION

Mitotic poisons are known to affect mitosis in one or both of two different ways, either by preventing cells from entering division or by arresting mitosis in one or more of its phases. The mitotic index, used in the present work, defined as the number of cells in division per 1000 cells scored, is not always an unequivocal expression of mitotic activity, since a lengthening of the interphase as well as a shortening of the mitotic period will produce a reduction in the mitotic index (Evans, Neary and Tonkinson, 1957). The considerable decrease in the mitotic index with the increased concentration (e.g. Fig. 1, 4, 6 and 7) of the substances tested, however, makes it unlikely that the effect of the materials was due to a shortening of the mitotic period. Therefore it is concluded that the materials described in the present work reduce the number of cells entering prophase or completely block the cells in interphase and belong to the group of inhibitors of preprophase or earlier stages, which include a large number of substances with—probably—a widespread mode of action at different stages during interphase. It is known that many preprophase inhibitors seem to affect the cell at the stage immediately before the visible prophase. Had this been the case for materials tested, a marked decrease in the mitotic index would have been found already after 3 hours' treatment provided it was assumed that the active material penetrated the cell membrane sufficiently rapidly. Since the mitotic activity after 3 hours appeared only slightly different (Fig. 1, 2 and 8) from that in the controls, the materials most likely affect the interphase at an earlier stage. It should be borne in mind, however, that the present materials, which are of high molecular weight, may penetrate the cell membrane slowly and thereby making the effect less pronounced after 3 hours.

Materials IV and VI (from blood) and material V (from liver) arrest mitosis in HeLa cells, but not in Chang liver cells at the concentrations used (Fig. 5 and 8). However, material I prepared from liver inhibits mitosis in both types of cells (Fig. 1 and 3). The difference between materials I and V, both isolated from liver, is, as far as the effect on Chang liver cells is concerned, somewhat surprising. The two materials differ, however, in another way as they are prepared by alternative methods. Material I contains both dialysable and non-dialysable material whereas material V only contains the latter, and the difference in behaviour towards Chang liver cells could be referred to this variation in composition.

The analytical data (Table I) demonstrate that the main part of the materials consists of polypeptides, indicating that the substance responsible for the mitotic inhibitory effect might be a polypeptide (Dedichen, Laland, Laland, Voss, 1962). It must, however, be borne in mind, that the substance responsible for the inhibitory effect could also be a substance of low molecular weight attached to a polypeptide, such as the fluorescent component present in these materials (Laland, Dedichen, Laland and Thorsdalen, 1962).

It is known that high molecular substances such as mucopolysaccharides are mitotic inhibitors. Thus, Lippmann (1957) found, when injecting heparin, a highly significant decrease in the mitotic index in the Ehrlich ascites tumour *in vivo*. Most work done on these substances seems to indicate a poisoning of metaphase (Biesele, 1958). Bacterial polysaccharides have been found to exhibit a heparin-like effect on the gelation before spindle formation (Heilbrunn and Wilson, 1950). Furthermore, *E. coli* lipopolysaccharide has been found (Murphy and Wisner, 1962) to exert a cytotoxic effect on cells *in vitro* characterized by nuclear changes. In all experiments presented in this work with materials both from liver and blood, occasional abnormal mitotic configurations were observed, but in no case with higher frequency for cells treated with materials than for controls. After 24 hours' treatment a slight granulation of the nuclei appeared when concentrations high enough to produce complete arrest or almost complete reduction of the mitotic activity were used. The effect of the muco- and lipopolysaccharides on the mitotic activity of cells is therefore different from those described in the present work. In further contrast to the bacterial lipopolysaccharides the present materials are not pyrogenic (Dedichen, Laland, Laland and Voss, 1962).

It is interesting that materials I, IV, V and VI also interfere with the growth of vaccinia virus *in vivo* (Dedichen, Laland, Laland and Voss, 1962). Whether or not the antiviral and the antimitotic effect is due to the same substances cannot be decided at the present time.

It is difficult to decide whether there are one or several active substances and if these are identical in the preparations from liver and blood. We are inclined to think that the materials from liver and blood contain the same active substances.

It is tempting to speculate that this substance or group of substances is generally distributed in cells and that its cellular concentration could play some role in controlling the mitotic activity of mammalian cells.

In connection with the present work it is interesting to note that liver has been examined as a source of growth-interfering substances from 1937 and onwards (Rohdenburg and Nagy, 1937; Marshak and Walker, 1945; Saetren, 1956; Stich and Florian, 1958; Herbut and Kraemer, 1960). Furthermore it is interesting that Japanese workers (Suzuko, 1959; Nakahara and Fukuoka, 1961) who have followed a line of research other than ours, have obtained results which are somewhat similar. The Japanese work came to our knowledge during the completion of this paper.

It is a pleasure to thank Dr. Reidar Eker, director for the Norwegian Radium Hospital, for his interest and support in this work.

One of us (J. D.) would like to thank The Norwegian Research Council for Science and the Humanities and the Norwegian Cancer Society for financial support. A grant from the Norwegian Cancer Society (R.O.) is gratefully acknowledged.

### SUMMARY

The effect of several materials prepared from ox liver and blood on human cells grown *in vitro* has been studied. The mitotic index has been used as a measure of mitotic activity. Two materials prepared from liver by alternative methods arrested completely the mitotic activity of HeLa cells after 24 hours. One of these inhibited also the mitotic activity of Chang liver cells.

Materials prepared from blood were also found to inhibit mitosis in HeLa cells after 24 hours. This preparation in the concentrations used had no effect on Chang liver cells.

It is concluded that the materials exert their effect during interphase.

### REFERENCES

Biesele, J. J.—(1958) 'Mitotic Poisons and the Cancer Problem', Amsterdam, Netherlands (Elsevier Publishing Company).
Dedichen, J., Laland, P., Laland, S. and Voss, J.—(1962) *Acta med. scand.*, **172**, 121.
Evans, H. J., Neary, S. J. and Tonkinson, S. M.—(1957) *J. Genet.*, **55**, 487.
Gornall, A. G., Bardawill, C. S. and David, M. M.—(1949) *J. biol. Chem.*, **177**, 751.
Heilbrunn, L. V. and Wilson, W. L.—(1950) *Science*, **112**, 56.
Herbut, P. A. and Kraemer, W. H.—(1960) *Amer. J. Path.*, **36**, 105.
Laland, P., Dedichen, J., Laland, S. and Thorsdalen, N.—(1962) *Acta med. scand.*, **172**, 117.
Lippmann, S. M.—(1957) *Cancer Res.*, **17**, 11.
Marshak, A. and Walker, A. C.—(1945) *Amer. J. Physiol.*, **143**, 226.
Murphy, W. H. and Wisner, C.—(1962) *J. Bact.*, **83**, 649.
Nakahara, W. and Fukuoka, F.—(1961) *Gann*, **52**, 197.
Puck, T. T., Cieciura, S. J. and Fisher, H. W.—(1957) *J. exp. Med.*, **106**, 145.
Rohdenburg, G. L. and Nagy, S. M.—(1937) *Amer. J. Cancer*, **30**, 335.
Saetren, H.—(1956) *Exp. Cell Res.*, **11**, 229.
Stich, H. F. and Florian, M. L.—(1958) *Canad. J. Biochem. Physiol.*, **36**, 855.
Suzuki, S.—(1959) *Jap. J. exp. Med.*, **29**, 341.